# Constructal approach to cell membranes transport: Amending the ‘Norton-Simon’ hypothesis for cancer treatment

**DOI:** 10.1038/srep19451

**Published:** 2016-01-29

**Authors:** Umberto Lucia, Antonio Ponzetto, Thomas S. Deisboeck

**Affiliations:** 1 Dipartimento Energia, Politecnico di Torino, Corso Duca degli Abruzzi 24, 10129 Torino, Italy; 2 Department of Medical Sciences, University of Torino, Corso A.M. Dogliotti 14, 10126 Torino, Italy; 3 Department of Radiology, Massachusetts General Hospital and Harvard Medical School, Charlestown, MA 02129, USA; 4 ThinkMotu LLC, Wellesley, MA 02481, USA

## Abstract

To investigate biosystems, we propose a new thermodynamic concept that analyses ion, mass and energy flows across the cell membrane. This paradigm-shifting approach has a wide applicability to medically relevant topics including advancing cancer treatment. To support this claim, we revisit ‘Norton-Simon’ and evolving it from an already important anti-cancer hypothesis to a thermodynamic theorem in medicine. We confirm that an increase in proliferation and a reduction in apoptosis trigger a maximum of ATP consumption by the tumor cell. Moreover, we find that positive, membrane-crossing ions lead to a decrease in the energy used by the tumor, supporting the notion of their growth inhibitory effect while negative ions apparently increase the cancer’s consumption of energy hence reflecting a growth promoting impact. Our results not only represent a thermodynamic proof of the original Norton-Simon hypothesis but, more concretely, they also advance the clinically intriguing and experimentally testable, diagnostic hypothesis that observing an increase in negative ions inside a cell *in vitro*, and inside a diseased tissue *in vivo*, may indicate growth or recurrence of a tumor. We conclude with providing theoretical evidence that applying electromagnetic field therapy early on in the treatment cycle may maximize its anti-cancer efficacy.

Recently, it has been highlighted how mechanical phenomena are fundamental in any biological process at the organ, tissue, and cell levels as well as in biomolecular motors, with consequences also on genes, such as c-MET, TP53, etc., for example^1^. Moreover, increased extracellular matrix stiffness has been shown to induce expression of the microRNA miR-18a in breast tissue and this miRNA targets the tumour suppressor PTEN^1^. Of course, it is very difficult to describe carefully what happens at molecular and cellular levels if only one single process is considered, because life is a non-selective process with a cooperative superposition of processes, interrelated with one another. Consequently, a new biomedical engineering approach has been developed based on the well-known mechanical concept of open systems, an approach that obtained a great number of results in biomedicine^2^. This science, named mechanobiology, is an interdisciplinary field between the mechanical sciences and biomedicine. It is based on the experimental results that external mechanical forces in cells or tissue environments determine some physiological changes and even diseases. The fundamental result is represented by the explanation of the molecular mechanisms of mechanotransduction, by which cells sense and respond to biomechanical signals and convert them into biochemical signals^2^. Indeed, a great number of receptors, such as ECM molecules, transmembrane proteins, cytoskeleton, nuclei, and lipid bilayer, are sensitive to mechanical stimuli and react to them through inside-outside and outside-inside transduction.

Hence, many processes in cells are related to nano-mechanical properties of their membranes^3^,^4^. Consequently, chemotherapeutic drugs often prove more effective in monolayer culture studies than in the human body later on, due to their diffusion limitations across cell membranes *in vivo*. In (pre-angiogenic) malignant tumors, *in vivo*, the following three cell phenotypes are usually observed^5^:

1. proliferating cells near the border of the tumorous micro-mass or spheroid, where nutrient flows allow rapid cell metabolism and division;
2. quiescent cells towards the inside of the spheroid, where nutrient flows are not sufficient for proliferation but still allow cell survival, hence a reversible state;
3. Apoptotic cells (and eventually necrotic tissue) near the interior of the spheroid, where nutrient flows are not sufficient to sustain metabolism.

*In vitro*, 3-D-tissues with heterogeneous microenvironments can be simulated to some degree by using multicellular tumor spheroids^6^,^7^. Here, the role of acidity on cancer cell growth and invasion was investigated in both vascular and avascular tumors^8^ as was drug diffusion and the consequent effect of drug-induced cell death^9^,^10^,^11^.

Moreover, the physiological stimuli are seldom dynamic, depending on the magnitude, frequency, and duration of external perturbations. On the base of systems biology, the engineering approach to cell behaviour can develop the analysis of the bio-molecular transmission of the external signals in relation to their ability to alter the structure, function, and dynamics of a cellular system^2^. In this context, it is useful to consider two recent and seemingly disparate approaches: the aforementioned tumor spheroid model^5^ and the irreversible thermodynamic analysis of biosystems^12^,^13^,^14^,^15^,^16^,^17^. The tumor spheroid model introduces the concept of diffusion limitations of spatial nutrient gradients, which impact the local energy metabolism and affect overall spheroid physiology^5^. Indeed, cancer cells consume more glucose than normal cells under identical conditions^18^ because cancer cells oxidize a portion of the glucose; they convert it to lactate, explained by the saturation of the tricarboxylic acid (TCA) cycle, as it was highlighted by studying interstitial fluid^19^. Moreover, high lactate and low oxygen concentrations have been shown, *in vivo*, to be related to the increase of metastases and cancer recurrence, and, as a consequence, to a reduction in patient survival^20^,^21^,^22^,^23^.

In cells, many processes such as replication, transcription and translation need to convert molecular binding energy, chemical bond hydrolysis and electromagnetic gradients into mechanical work. This mechanical work is related to conformational changes and displacements^24^. Moreover, the implications of the mechanical analysis of DNA have been analysed in relation both to biological motors and to the design of synthetic nano-scale machines^25^. The biomechanical analysis of DNA has pointed out the connections among forces, thermodynamics, nano-mechanical and electromagnetic behaviour of biological structures and kinetics^25^,^26^. Furthermore, cell surface modifications were highlighted to enhance the therapeutic potential of cell products for medical therapy, such as, for instance, in the successful design of new drug carriers to overcome delivery barriers^24^. Consequently, the analysis of the bio-mechanical properties is important and it can further be improved by introducing a new, recently proposed thermodynamic analysis of the transport phenomena^12^,^13^,^14^,^15^,^16^,^17^ in the conventional mechanochemical approach to biosystems; indeed, this thermodynamic approach has highlighted both the fundamental role played by molecular machines in transport phenomena and also the consequences of these phenomena for cell metabolism and resulting cell behaviour.

In this paper, we wish to improve this mechanobiology perspective by introducing a comprehensive thermodynamic approach, based on the transport phenomena across the cell membranes. Consequently, we suggest a thermodynamic explanation - based on the constructal law and entropy generation analysis of the cellular system - for some biomedical observations. To do so, in Section 2 we summarize some of the experimental results presented in the literature, while in Section 3 we summarize the usual bioenergy analysis of the cell system based on the classical spheroid model, here generalized by introducing a mean sphere radius. In Section 4 we develop this new concept, named bioengineering thermodynamics, a thermodynamic approach based on fluxes, a constructal approach to bio-systems, which is clearly useful to introduce some new approaches to medicine, as the newly obtained relation between apoptosis/mitosis rate can show. Finally, Section 5 summarizes the findings and, to illustrate their relevance in practice, we put them in perspective to the so-called Norton-Simon hypothesis, with potentially significant implications for anti-cancer therapy.

## Experimental Results

Ion channels and pumps generate bioelectricity. Indeed, ion flows cause variations in the bioelectrical potential at the membrane level because this last quantity can be evaluated by using the Goldman–Hodgkin–Katz equation^27^,^28^:


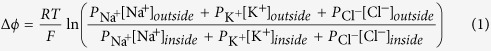


where *P* is the permeability of the ion, [A] means concentration of the A-ion, *R* is the ideal gas constant (8.314 J mol^−1^K^−1^), *T* is the temperature, and *F* is the Faraday constant (96.485 × 10^3^ A s mol^−1^). Indeed, relation (1) points out how the membrane potential can be changed by alterations in the conductance of one or more ions. The ion channels and transporters provide different permeability to distinct ions, such as Na^+^, K^+^, Ca^2+^, and Cl^−^. As a consequence of the asymmetry in these ion distributions, a membrane potential exists between the cytoplasm and the extracellular environment. It is expressed relative to the extracellular environment and a cell depolarizes if the membrane potential is relatively less negative, and *vice versa*^29^. It is noteworthy that, for example, the V-ATPase pump can change the transport of H^+^ and, as a consequence of the Cl^−^-H^+^ coupled transport, it can change the Cl^−^ transport. Considering the relation:


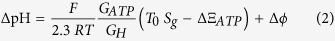


where *G* is the flux, Ξ is the Gibbs function, *H* means hydrogen ion (proton), *ATP* means ATP, 0 refers to environment and *S*_*g*_ to entropy generation, it is possible to state that both the membrane potential and the pH are changed by any alteration of the V-ATPase. And this happens also for the other membrane pumps and ion channels. So the change of inside/outside membrane pH and transmembrane electric potential are related, and they lead to consequences in the behaviour of the cells. On the other side, changes in cell behaviour must determine variation in the inside/outside membrane pH and transmembrane electric potential. All these phenomena can be regulated only by the energy, mass and ion transport across the cell membrane. We note that this, our thermodynamic result, obtained by theoretical considerations on fluxes and irreversibility, will need to be experimentally supported. As such, in this section we summarize the experimental results that confirm this result.

Bio-electricity is a definition of all the phenomena related only to the endogenous electric signaling based on ion channels or pumps across the cell membrane. It excludes the external electromagnetic fields, the ultra-weak bio-photon emission and the sub-organelle potentials^30^.

In this context, it was highlighted that transepithelial electric fields regulate cell migration, orientation and growth^31^,^32^. Recently, new aspects of bio-electricity have been related to the regulation of individual cell function, embryogenesis and regenerative repair of complex structures^30^,^33^,^34^ in non-neural cells and cancer. Moreover, in human mesenchymal stem cells^35^, cardiomyocytes^36^, vascular muscle^37^, embryonic stem cells^38^, myoblasts^39^, the control of precursor differentiation^40^ in the developing nervous system and heart, etc., it was shown that differentiation and proliferation are controlled by changes in the membrane’s electrostatic potential.

These experimental reports support our thermodynamic results. Moreover, considering the role of the electrostatic potential in regulating normal migration, differentiation, and proliferation, its control, or lack thereof, is fundamental for the development of cancer as well^30^. Notably, this result can be obtained simply by the control of the ion fluxes. Indeed, the voltage-responsive transduction mechanisms on the cell membrane allow bioelectric signals to regulate cell polarity. The cytoskeleton is one target of such signalling^30^. Also, asymmetric distribution of ion transporter proteins in the early blastomeres, and the related gradient drives unidirectional serotonin flow through cell fields with effects on the differential gene expression on the left versus right sides of the body^41^. For example, the V-ATPase pump for H^+^ or Voltage-gated calcium signalling as the transduction mechanism can be used to modify the upstream endogenous bioelectrical signalling as a response to physiological, transcriptional, and mechanical signals, while downstream of membrane voltage can determine the mRNA and chromatin modification levels. Transcriptional responses to depolarization include genes and other biomolecules in cells^34^. A number of transduction mechanisms have been highlighted to be capable to change the resting potential at the nucleus; example are the voltage-gated calcium channels^42^ or the voltage gradients among cells to move small signalling molecules such as serotonin^4^.

### Multicellular spheroid model

In this section, the multicellular spheroid model will be briefly summarized so that we can build on it for our thermodynamic approach to cell growth. This *in vitro* model is interesting because it represents a simple approach to extracellular metabolite concentrations as well as to intracellular metabolism related to the nutrient flows for energy (ATP) conversion in a cell^5^.

We consider a control volume, i.e. a definite volume, which contains *N* cells, constant in time. Inside the spheroid, the cell population is comprised of living and dead cancer cells. We indicate with *n* the total cell density, with *n*_*l*_ denoting the density of live cells and *n*_*d*_ that of dead cells. These densities are a function of time *t* and position **x** inside the control volume. For this control volume the mass balance equations can be written as^43^:


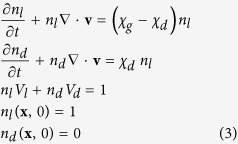


where **v** is the bulk convective velocity of the cell mass, *χ*_*l*_ is the cell growth rate, *χ*_*d*_ is the cell death rate, *V*_*l*_ is the living cell volume and *V*_*d*_ is the dead cell volume. Cell proliferation generates a volume increase, while cell death is the cause of the loss of volume; consequently, spheroid expansion is driven by the relative rates of cell growth and cell death^5^. The effect of the local volume alterations is a radial velocity gradient for the cell mass; it can be evaluated as^43^:


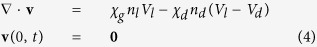


The spheroid growth rate can be obtained by evaluating the rate of the spheroid radius variation:


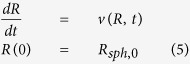


where *R*_*sph*,0_ is the initial spheroid radius, evaluated as^5^:


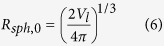


The metabolic model used here is based on the results of Venkatasubramanian *et al.*^5^, which introduces the assumption that tumor cells can consume three nutrients: glucose (*gluc*), oxygen (O_2_) and lactate (*lac*). The nutrient transport balance equations are then as follows:


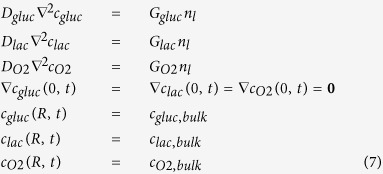


where *c* is the local nutrient concentration in the spheroid interior, *bulk* means the bulk nutrient concentration outside the spheroid, *D* is the nutrient diffusion coefficient, *G* is the nutrient uptake rate, a function of the vector **c**, containing all the nutrient concentrations and related to the availability of each nutrient in the extracellular environment. The nutrient transport rates across the membrane represent the upper limits of the metabolic uptake rates, described by the Michaelis-Menten equations^44^:


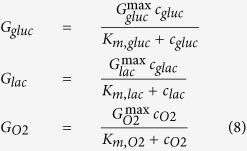


where *G*^max^ are the saturated uptake rates at maximum values, *c* is the concentration and *K*_*m*_ are the substrate concentrations at which the reaction rates result as^45^ *G*^max^/2. Starting from these relations the cell growth rate and the death rate, respectively, was suggested to be^5^:


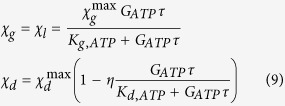


where *η* represents the basal survival rate, *K* is the maximum cell growth or death rate and *G*_*ATP*_ is the net local ATP production rate. In order to evaluate this last quantity, some considerations on the intracellular metabolism stoichiometry have been introduced^5^:

- 1. Cells consume glucose as soon as it is available; consequently:

- Glucose uptake is limited only by its transport across the cell membrane, evaluated in relation (6);
- Oxygen uptake is limited either by its own transmembrane transport or by transmembrane glucose and lactate transport.
- If the oxygen extracellular concentration is low, its transmembrane uptake is limited.
- When the glucose and lactate concentrations are low, the oxygen uptake rate is limited by the intracellular metabolism stoichiometry;
- The conversion of 1 glucose molecule into 2 lactate molecules produces 2 ATP molecules;
- The complete oxidation of 1 glucose molecule produces between 30 to 38 equivalent ATP molecules;

- 2. The electron transport system converts all reductive equivalents generated by glycolysis (NADH and FADH) into ATP; consequently:

- Oxygen is consumed by the electron transport system with a consequent production of water;
- Each oxygen molecule consumed in complete oxidation of pyruvate in the TCA cycle produces an equivalent of 5 ATP molecules;
- The excess glucose is converted into lactate;

- 3. Lactate can be consumed as a carbon source when the concentration of glucose is low and the concentration of oxygen is high; consequently,

- The rates of glucose and oxygen uptake determine the rate of lactate consumption and production, regulated by the intracellular reactions stoichiometry;
- Lactate is consumed and oxidized as a carbon source;
- The complete oxidation of 2 lactate molecules would generate up to 36 equivalent ATP molecules;

- 4. The Phosphate/Oxygen Ratio for NADH and FADH_2_ are 3 and 2; consequently:

- The conversion of 1 glucose molecule into 2 pyruvate molecules produces 8 molecules of ATP, while it consumes 4 of them;
- Glycolysis produces 2 ATP molecules and 2 NADH molecules, equivalent to 6 ATP molecules;
- The conversion of 1 lactate molecule into 1 pyruvate molecule produces 1 NADH molecule, equivalent to 3 ATP molecules;
- The complete oxidation of 1 pyruvate molecule consumes 3 oxygen molecules and produces 4 NADH molecules, 1 FADH_2_ molecule, and 1 GTP molecule, equivalent to 15 ATP molecules.

Consequently, the net ATP production is obtained by the following chemical balance:


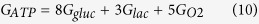


The approach considered is based on the assumption that cell growth and death rates can be obtained from a thermodynamic analysis of the cells’ energy balance of the local ATP generation rate.

### The thermodynamic approach

Cells are biological engines with specifically ordered chemical reactions. A part of their energy is lost as heat outflow and only the resulting products of biochemical processes are known, while any individual step is largely inaccessible. Consequently, as already detailed in Lucia *et al.*^17^ the study of cells can benefit from introducing the ‘black box’ model where we consider only the spontaneous cell flows.

Therefore, the spontaneous heat cell exchange represents the interaction or, here, the spontaneous communication between the cell and its environment^12^,^13^,^14^,^15^,^16^,^17^, i.e., the heat flow across the cells membrane. In particular now, considering that^46^:


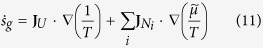


with **J**_*U*_ being the energy flow, *T* the temperature, 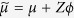 the electrochemical potential, *μ* the chemical potential, *z* the electric charge per unit mass, *ϕ* the electrostatic potential and **J**_*Ni*_ the molar flow (ions included), it follows that this heat flow is the consequence of irreversible processes within cells, and this is easily developed by using the Gouy-Stodola theorem, but also that any irreversible process inside the cell causes flows across the cell membrane. This entropy generation is related to the work lost for irreversibility and the temperature of the system’s environment. The fundamental quantity introduced to analyze this work lost is the total entropy generation, defined as:


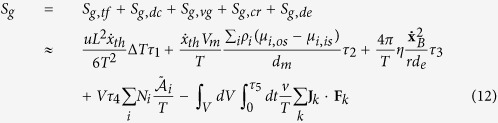


where:

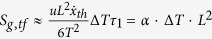
 is the entropy generation due to the thermal flux driven by temperature difference;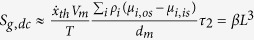 is the entropy generation due to the diffusion current driven by chemical potential gradients;
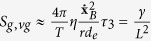
 is the entropy generation due to the velocity gradient coupled with viscous stress;
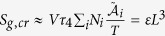
 is the entropy generation due to the electrochemical reaction rate driven by electrochemical affinity 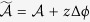, with 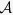 being the chemical affinity, related also to pH variation and the membrane’s electric field variation;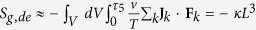 is the entropy generation due to dissipation caused by work through interaction with the environment; it depends on the kind of field considered;

with *L* introduced as a characteristic length, as is usually done for studying transport phenomena.

These relations have been recently evaluated by numerical approximations in Lucia *et al.*^17^, and, as a consequence of these results, the total entropy generation for a cell can be written as a function of the mean diameter, which leads to:


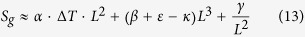


In order to evaluate the related growth of a cancer, it is necessary to develop some results obtained from the analysis of its geometry. Cells have a fractal geometry^47^,^48^,^49^, consequently, the cell mass increases as a function of a characteristic length and it can be evaluated as:


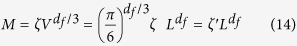


with^47^ 2 < *d*_*f*_ < 3 fractal dimension for a three dimensional approach and *ζ* and *ζ*′ being proportional constants. Higher fractal dimension results in larger organ size. The fractal dimension is related to the ratio between mitosis rate and apoptosis rate^50^:


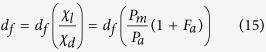


with *χ*_*l*_ the cell reproduction rate being constant and *χ*_*d*_ or cell death rate remaining constant, defined as^13^:


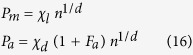


where *n* is the number of cells, *P* is the probability per unit time, *m* means mitosis, *a* stands for apoptosis, *F*_*a*_ is a dimensionless correction term which represents the relation between the cancer radius and a characteristic length of the volume, and it takes into account the finite size of the host^50^; *d* is a constant. Some numerical evaluation for *in vitro* cancer and a two dimensional approach is developed in Izquierdo-Kulich *et al.*^50^. In relation to these considerations and generalizing the approach used in Izquierdo-Kulich *et al.*^50^ from two to three dimensions it is possible to obtain the entropy generation due to affinity as a function of *χ*_*l*_ and *χ*_*d*_, and using equation (14) in relation to the mitosis and apoptosis probability^13^:


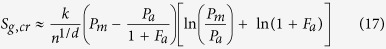


with *k* being constant. Now, considering relation (7) it follows that:


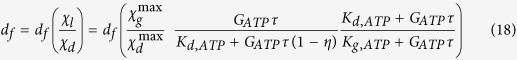



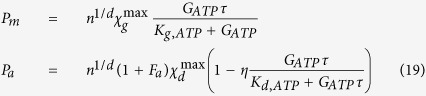



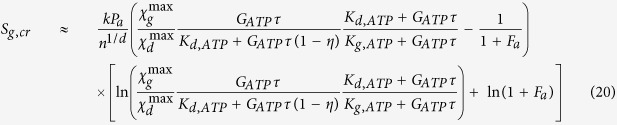


which means that the ATP production rate determines:

1. the fractal dimension of the cancer system;
2. the probability of mitosis and apoptosis;
3. the entropy generation variation due to chemical reaction.

But in relation to the entropy generation variation due to chemical reaction we can introduce some more considerations. Indeed, if we consider that a cancer (or a normal cell system) tries to obtain an optimization process to live, it is possible to state that its entropy generation variation is at a maximum if evaluated from the environment and a minimum if evaluated from the system^51^,^52^, so:


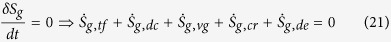


Here, we are analyzing the process related to the glucose, lactate and oxygen flows across the membrane, so, as a first approximation we do not consider entropy generation variation for the thermal flux driven by temperature difference and for the velocity gradient coupled with viscous stress, while we consider the possibility to use external fields to allow the cell processes. Consequently, we can obtain:


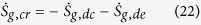


This last relation represents an important result because:

1. It relates the diffusion current to the chemical reaction when any external electromagnetic field is applied, in agreement with the stoichiometric constraints previously introduced;
2. It relates the diffusion current to the use of external electromagnetic fields which can allow or prevent the cellular biochemical reactions;
3. It suggests that external fields could be used therapeutically to control the energy conversion of the cells. In particular, in relation to cancer, this control could be used in support of the actual anticancer therapies;
4. The probability of apoptosis and mitosis is related to the entropy generation and to the nutrient flows, as is the cell system’s fractal dimension, consequently, the geometrical and growth evolution of cancer can be controlled by external use of fields, as suggested in Lucia *et al.*^17^;
5. The relations (9), (18), (19) and (20) highlight how the ion, mass and heat fluxes across the membranes are the first causes of all the processes inside the cells because these fluxes change the entropy inside the cells, with the consequence of changing the state of the cells themselves.

Finally, from the previous relation we can assess the ratio of the ATP flows vs. the one during mitosis as a function of the rate of cell reproduction and death. It follows:


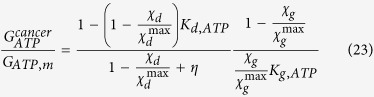


**Figure 1:**
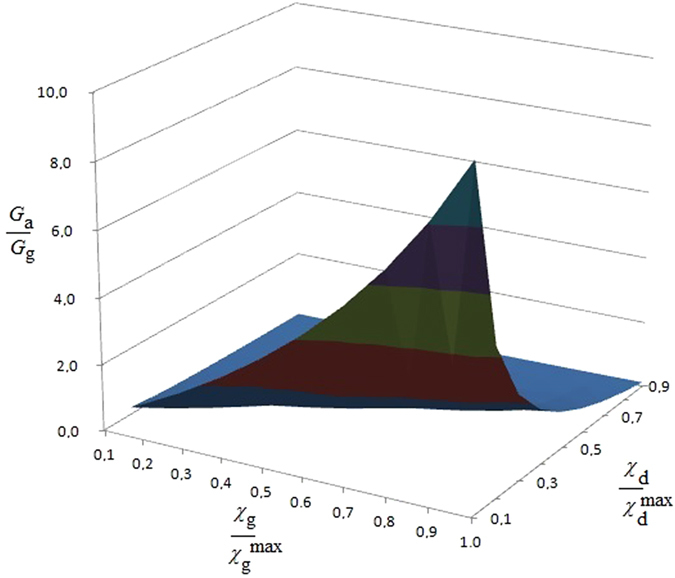
This figure represents, during mitosis, the ATP flow [mol s^−1^] for a tumor cell vs. the ATP flow [mol s^−1^] for a normal, non-cancerous cell as a function of the cell reproduction 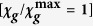 and death rates 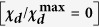, weighted on their maximum values. These last quantities are non-dimensional values representing the growth and apoptosis rate of cells within a range [0,1]. It can be seen that both, an increase in proliferation and a reduction in apoptosis trigger a maximum of ATP consumption by the tumorous cell.

The result is presented in Fig. 1: shown is on the y-axis the ATP flow [mol s^−1^] for a tumor cell vs. the ATP flow [mol s^−1^] during mitosis for a normal, non-cancerous cell as a function of the cell reproduction and death rates weighted on their maximum values. These last quantities are non-dimensional values representing the growth and death rate of cells within a range [0, 1]. The result shows that the ATP flow required by a cancer cell to maintain its maximum reproduction rate 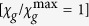 and minimum death rate 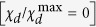 is 10 times that of a normal, non-cancerous cell. Since this result has been obtained considering the mass flow balance^10^, if we extend this concept to include all the chemical mass flow it could be even greater. This proves that oncogeneic transformation requires more energy than a normal cell, rendering it essential to satisfy this energy demand by increasing the mass flows of glucose and other nutrients to produce ATP. Consequently, controlling ions and mass flows across the membrane should present a powerful method to more effectively control the growth of cancer.

## Discussion and Conclusions

The study of mutations and the activation of oncogenes is fundamental to understand the basis of tumorigenesis^53^. The results obtained here suggest that these gene alterations could be the result of phenomena which can alter the flows across cell membranes, in agreement with the recent link obtained between oncogenic miRNA induction, tumour mechanics and tumour suppressors^54^. Similarly, it could also be argued, that random (or induced) gene mutations/alterations can hit protein-protein pathways that in turn can alter flows across the membrane. From a thermodynamics point of view the fundamental result is the link between the two related phenomena and their quantitative description, but which of the two biological phenomena is the underlying cause cannot be identified at present. To appreciate how all this relates to several open problems in bioelectricity^30^ some considerations must be developed.

First, to understand the regulation of single-cell behaviour and multicellular pattern formation, one should consider not only gene and protein profiles. Rather, ion channels and gap junctions are essential molecular elements of such circuits; however, bioelectrical signalling has its own unique dynamics that will become increasingly tractable with the development of new technology specifically targeting stable trans-membrane potential states. The existence of bioelectric signalling among most cell types, not only neurons, suggests a wide field of applicability of these electro-magnetic signals. Still, largely unclear remain the mechanisms by which cells compare bioelectric states across distances, and missing are additional molecular details of the interactions of bioelectrical signals with chemical gradients, and the development of quantitative models of bioelectric circuits that store stable patterning information during morphogenesis. This not withstanding, the control of bioelectric circuits could have important implications for bioengineering, regenerative medicine, and cancer biology research alike.

Here, we provide a new, thermodynamic theory that helps explain the effects of energy, mass and ionic flows across cell membranes. Intriguingly, this concept represents a theoretical explanation of several experimental results seen in biomedicine and bioelectricity.

We note that one context in which bioelectric and genetic state information can diverge is cancer. For instance, a metastatic phenotype can be induced in genetically normal melanocytes by depolarization of somatic cells^41^. This effect is not independent from the cell interaction with the microenvironment, hence, conceivably, some bioelectric states of the surrounding normal cells can trigger metastatic behaviour in neighboring cancer cells.

Conversely, the formation of tumours by human oncogenes (MET and KRAS, for example) can be suppressed by artificially preventing the depolarization that occurs during oncogenic transformation^55^. As such, improving our knowledge about the bioelectric processes involved, and using the theory put forward here, may well lead towards new and more effective anti-cancer strategies, as discussed in more detail in Section 5.1 below.

This new approach is based on studying the irreversibility of the cell processes, which is strictly related to the interaction between internal cell reactions and membrane transport phenomena. The fundamental difference is that transport phenomena can be analysed by studying the cell environment, while the interior of the cell is inaccessible without perturbations of the cellular biological processes. So, the analysis of cell environment and membrane flows should provide a more promising route to better understand cell behaviour.

Living cells are separated from their environment by the lipid bilayer membrane, which presents a different concentration of specific ion species on both sides. As a consequence, a charge separation across the membrane is generated by the electro-diffusion of ions down their electrochemical gradient. These ions move into a negative (inside the cell) membrane potential of around −70 mV to −100 mV. The hydrophobic component of the lipid bilayers behaves as a capacitor dielectric, which maintains the ionic gradients across the membrane; in some instances, the action of ATP-driven ionic pumps supports this effect by separating the charges. The membrane thickness is around 27 × 10^−10^ m, so the resulting membrane potential approximates 10^7^ V m^−1^. The cell function is regulated by the membrane proteins that are sensitive to an electric field; indeed, changes in the electric field are transduced into a conformational change that accomplishes the function of the membrane protein with consequences for the regulation of cell functions. The charged species, their arrangements, the local field strength, charges and dipoles disposition and movements can vary with the result of changing the electric field which is transduced into a conformational change related to the protein functions themselves. Indeed, molecules as Aspartatic acid, Glutamic acid, Arginine, Histidine and Tyrosine can re-orientate in the field as has been proven for voltage-gated ion channels^56^. Moreover, changes in an electric field could move free ions as a result of a conformational change, as it happens in the case of the Na^+^-K^+^ pump^56^.

### Practical implications

The considerations above suggest that control and regulation of the membrane’s electric field could represent a new approach to therapies against diseases such as cancer. Intriguingly, our result confirms the well-known Norton-Simon hypothesis, which states that “Therapy results in a rate of regression in tumor volume that is proportional to the rate of growth that would be expected for an unperturbed tumor of that size”^47^. Indeed, our results allow us to *prove analytically* that Norton-Simon is not just a hypothesis but a biophysical consequence of the maximum rate of flux of ATP for apoptosis/flux of ATP for mitosis. Note that, this Norton-Simon-supporting result is related to the expected growth or apoptosis only when these relative rates are 1, as represented in Fig. 1.

To understand how to control the fluxes across the membrane we introduce a simple model of membrane transport. To do so, we consider the concentration of the ions on the opposite sides of the membrane^57^:


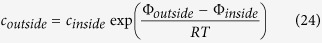


where *c* is the molar concentration of the chemical species, summarized in Table 1, *R* is the universal constant of gas, *T* is the temperature and Φ is the electric potential energy. As a consequence of this concentration difference the cell can move the ions, and change the pH inside and outside its membrane. The ion drift velocity *v*_*drift*_ across the cell membrane can be obtained by using the classical kinetic theory^58^ as:


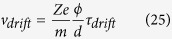


where *Ze* is the electric charge of the ion, *m* is the ion mass, *ϕ* is the electric potential across the membrane, *d* is the length of the membrane and *τ*_*drift*_ is the mean time between two collisions^57^:


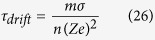


where *σ* is the electric conductivity, set to be around^57^ 2 Ω^−1^m^−1^. Consequently, an electric current *I* occurs for each ion *i* = H^+^, Na^+^, K^+^, Ca^2+^, Cl^−^, Mg^2+^:


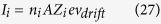


where *A* is the mean surface area of the membrane. Now, considering the equivalent RC electric circuit for a membrane it is possible to state that the resonant frequency for such a circuit results in (2π*RC*)^−1^, where *R* is the electric resistivity for the ion considered and *C* is the membrane capacity. It follows, that if we want to control the cross-membrane flux we must impact the current. The easier physical way to interact with a current is to use an electromagnetic wave of the resonant frequency for the membrane, in relation to the ion considered, with its amplitude being related to the entropy generation as just obtained in our previous papers^14^,^17^.

It has been proven that tumours present a state of growth instability, a state in which a small tumor cell fraction exhibits a higher proliferation rate than the parent strain. In fact, it has been highlighted that^59^:

- if this instability precedes the onset of chemotherapy treatment, the slope of the linear increase of the drug concentration for the standard “Norton-Simon late intensity schedule” changes;
- the initial value of the dose strongly depends on the balance of the tumor cell population and on their growth rates;
- if the instability trails the initial treatment, the effective chemotherapeutic drug concentration changes.

To therapeutically target a solid cancer, diagnostic visualization is key and its technical limit in patients is currently assumed to be around 5 mm in diameter. However, at this stage, the cell growth curve starts to plateau and the cell population is highly heterogeneous with the consequence that chemotherapy efficacy can no longer be expected^60^.

Conventional human anti-cancers therapies are not sufficiently selective to determine dose schedules that maximize the benefit/toxicity ratio^61^. In order to approach this topic, Simon and Norton used clinical and laboratory experiences to obtain a phenomenological law for evaluating the cytotoxic chemotherapy effects on tumor size in relation to the tumour growth dynamics. The analytical expression of what became the Norton–Simon hypothesis is that “a tumor’s size at a given time point depends on the integrated drug effect during the course of treatment up to that time”^61^.

Our thermodynamic approach represents a biophysical and biochemical proof of this hypothesis. Indeed, we relate the effects of the therapies to the ability to control the mass, energy, ions and, also, chemicals across the membrane. Intriguingly, relation (23) can be extended to every chemical species, which crosses the membrane. It allows us to state that there exists a maximum value of the quantity, which can cross the membrane due to the finite drift time as evaluated in relation (26). Relation (27) allows us then to state that it is possible to control these fluxes. Consequently, we can improve the Norton-Simon hypothesis by amending it with a thermodynamic theorem: chemotherapy results are related to the drift time of the drug used while the rate of regression in tumor volume is proportional to the possibility to control the drug flux across the membrane and it determines the rate of growth for an unperturbed tumor of that size which is related to the integrated drug effect during the time of treatment.

Indeed, considering the relation between the growth in cancer mass and the energy lost, measured as a function of the temperature variation between the cancer cell and its environment, obtained in a previous paper^15^, it is possible to write the energy lost as:


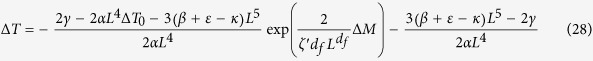


where *ζ*′ is a constant, *T* is the temperature, proportional to the heat wasted by the system and, consequently, proportional to the energy exchanged by the system itself with its environment, and Δ*M* is the mass variation for cancer growth. This general bioenergetic equation can be written in relation to the analysis here developed, i.e. the electrochemical reactions, and considering that^15^ 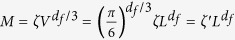, it follows that, in absence of any external field, equation (28) becomes:


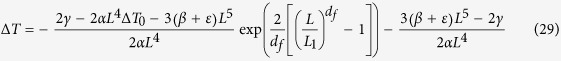


where *L*_1_ is the mean diameter of the cell at the start of the observation, i.e. the initial tumor size. We focus on the control of the fluxes and, consequently, on the interaction with the electrochemical and thermophysical properties of the cell membrane. It is related to the values of the coefficient 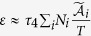 where 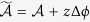 is the electrochemical affinity and 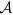 the chemical affinity, related also to pH variation and the membrane’s electric field variation; hence, this coefficient is fundamental in our analysis. It is possible to highlight how this coefficient is related to:

1. the proper time *τ*_4_ of the process, as previously discussed in Section 4. This time represents the biomedical inertia of the system in relation to chemical reaction rates: the smaller the value, the slower are the chemical therapeutic effects;
2. the electrochemical affinity, i.e. to the chemical affinity, related also to pH variation and to the membrane’s electric field variation;
3. the temperature of the system;
4. the numerical quantity of cancer cells.

The Norton-Simon hypothesis considers only the time and the number of cells. Our model agrees with Norton-Simon, but beyond, it improves it by adding other information about the system such as temperature, cell number density and electrochemical affinity. Our approach can therefore modify this important anti-cancer hypothesis into a thermodynamic theorem in medicine.

Moreover, we, and others, have previously stated that an external magnetic field could help control cancer growth^62^. In order to verify this statement, we consider relation (29) in the presence of an external magnetic field; this leads to:


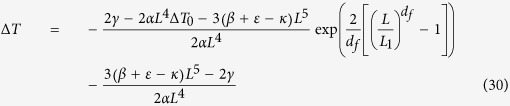


**Figure 2:**
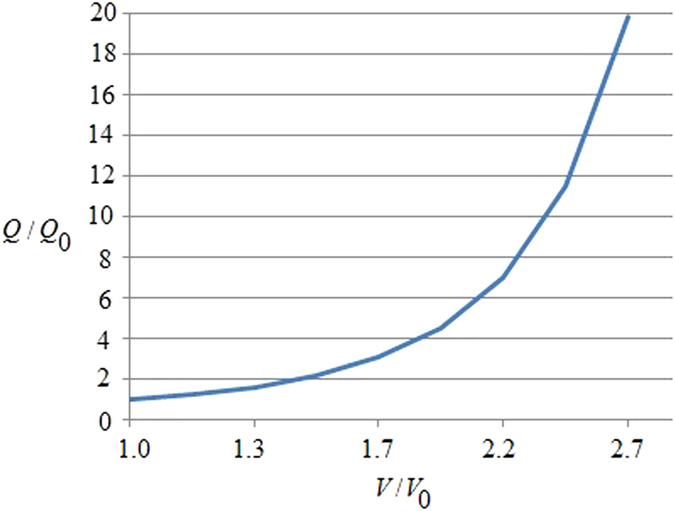
This figure represents the ratio of energy consumption vs. the volumetric growth of cancer. The *y*-axis is the ratio between the energy used by a cancer cell, *Q*, related to the energy used by a normal, non-cancerous cell, *Q*_0_. The *x*-axis is the ratio between the volume of cancer *V* related to its initial value *V*_0_ at time 0. This result has been obtained by using Eq. (29). Note that a cancer cell and therefore tumor tissue consumes much more energy than normal tissue; moreover, the energy required by the cancer is proportional to the exponential tumor growth rate.

**Figure 3:**
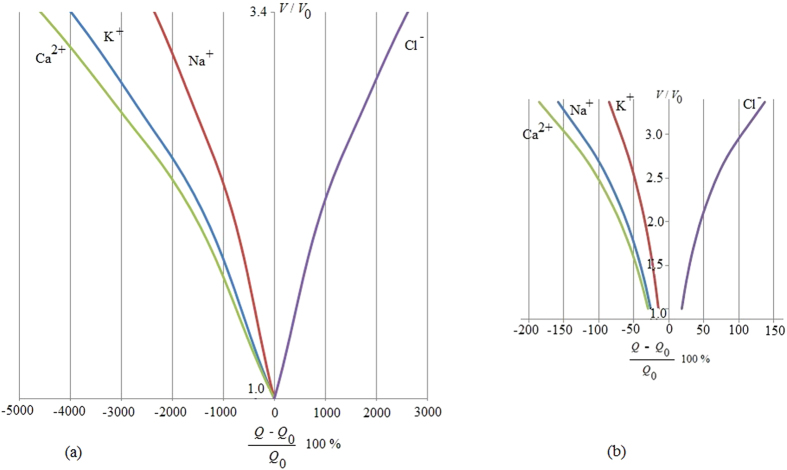
The figure represents the volumetric growth of cancer tissue under a magnetic field (50 μT, 40 Hz) in relation to the energy used by the cancer with regards to the ion involved. The *y*-axis represents growth, i.e. the ratio between the volume of cancer *V* related to its initial value *V*_0_. The *x*-axis displays the ratio (in percentage) in energy consumption used for growth between a cancer cell and a normal, non-cancerous cell; here, *Q* is the energy used by the cancer cell while *Q*_0_ is the energy consumed by the normal cell; minus (−) and plus (+) are related to the motion of the ions in relation to the membrane’s bioelectric field; consequently, in the case of Cl^−^ inflow the cell gains energy, which in turn can be used for continued growth, while, to process Ca^2+^, K^+^ and Na^+^ inflow, the cell loses energy and thus growth potential as it works against the membrane potential to sustain the influx of these ions. As the tumor grows, all ion fluxes change but their percentage changes are much larger when the tumor is exposed to the magnetic field in (**a**), reflecting a much more pronounced consumption of ATP to process these fluxes. The inhibitory effect of the magnetic field is evident as the tumor, to achieve the same volumetric growth, is forced to consume much more energy in (**a**) than in (b) when it is not exposed to a magnetic field. Still, since Cl^−^ influx increases also in (**a**), particularly at later growth stages, this further supports the argument that magnetic field therapy may lose efficacy eventually (which is being explored in more detail in Fig. 4).

**Figure 4:**
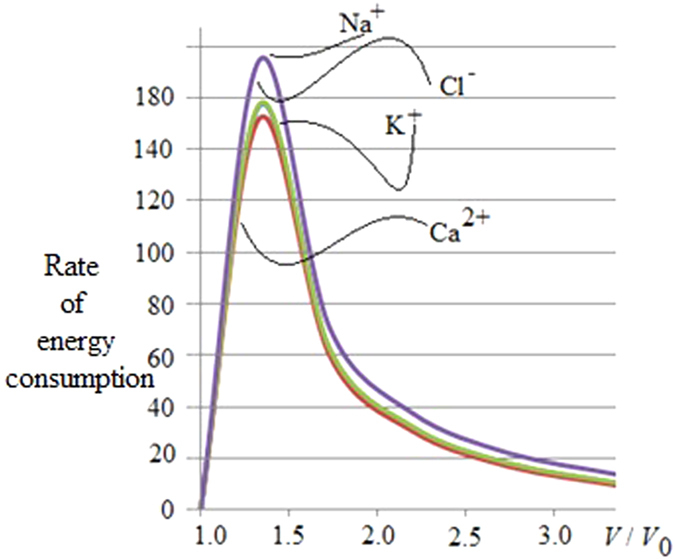
The figure represents the ratio between the energy consumption of a cancer cell inside the magnetic field (50 μT, 40 Hz) and outside of it; the *y*-axis is the quotient of the *x*-axis of Fig. 3(a,b), with the *x*-axis again depicting the volumetric increase of the resulting tumor. It is evident that tumor tissue consumes much more energy at the early growth stages, which supports the temporary effectiveness of the magnetic field therapy that rapidly diminishes at later, more advanced tumor growth stages, hence advocating for applying this therapeutic modality early on in the treatment cycle (see also Fig. 3). While this treatment response is reflected in the flux patterns of all four ions, it is conceivable to pinpoint the maximum benefit of the magnetic field to the largest ratio between the Na^+^ and Ca^2+^ fluxes which may have diagnostic value (note that the Na^+^ and Cl^−^ fluxes are superimposed in the figure). As to the tumor, based on Eq. (16) and Fig. 1, we argue that rapid cancer cell proliferation dominates early on whereas marked apoptosis impacts the result at later stages. (Note: due to the current setup, we are unable to assign a distinct weight to the relative contributions to [total] energy consumption of intrinsic tumor growth vs. therapeutic impact of the magnetic field, an area that we would like to explore in future works).

From this relation it follows that there exists a different thermal response of the system in relation to the application of an external magnetic field; this could be exploited to optimize anti-cancer therapies, as presented in Figs 2 and 3. Figure 2 represents the evaluation of relation (29) which not only yields that a cancer cell, and therefore tumor tissue, consumes much more energy than the normal tissue but also that this energy required by the cancer is proportional to the exponential tumor growth rate. In the presence of a magnetic field, as depicted in Fig. 3a, the tumor is forced to consume much more energy than without its therapeutic impact (3b); intriguingly, this in turn should have implications for tumor angiogenesis and/or blood flow demand, which then may confirm a role for adjuvant anti-angiogenesis schemes. As the tumor grows, all ion fluxes change but their percentage changes are much larger when the tumor is exposed to the magnetic field in (3a), reflecting a much more pronounced consumption of ATP to process these fluxes. These figures have been obtained by using relation (30). Note also how the different ions have distinct effects on the tumor’s use of energy: The positive ions lead to a decrease in the energy used by the tumor (*x*-axis), suggesting a growth retardant if not even inhibitory effect while negative ions apparently increase the cancer’s consumption of energy. Consequently, one could argue that observing an increase in negative ions may indicate growth or recurrence of a tumor. We caution however that, while this novel, entropy-based approach allows us to highlight the interactions between a biosystem and its environment, it does not yet shed light of cause and effect. Still, in our study, a more effective ion is Ca^2+^, which may indicate that a therapeutic modulation of calcium ions could yield an inhibitory effect on cancer growth, a notion that is supported by several experimental findings^63^,^64^,^65^,^66^. These last results confirm the Norton-Simon hypothesis and our own thermodynamic results. Indeed, these simulations prove how ion flow control across the membrane can lead to a different response by the cancer, thus potentially signalling a novel avenue for cancer research and ultimately, for therapy. This hopeful vision is supported by Fig. 4, which shows the ratio between the energy consumption by a cancer inside the magnetic field (50 μT, 40 Hz) and outside of it. It further highlights how the magnetic field exerts resistance against the growth of cancer particularly at the early stages of tumor growth, when rapid cell proliferation dominates, while its effectiveness tampers off later on which seems to support advocating for magnetic field therapy early on in the treatment cycle, as a first line modality rather than as a last resort.

We therefore conclude that this new thermodynamic approach, based on the constructal law^51^,^52^ and the entropy generation approach^67^, not only has the potential to advance biosystems engineering^68^,^69^ but also that it has significant practical applicability for a variety of important medical challenges, including advancing cancer treatment^70^,^71^,^72^.

## Additional Information

**How to cite this article**: Lucia, U. *et al.* Constructal approach to cell membranes transport: Amending the ‘Norton-Simon’ hypothesis for cancer treatment. *Sci. Rep.* **6**, 19451; doi: 10.1038/srep19451 (2016).

## Figures and Tables

**Table 1:** Typical ion concentrations and Nernst potential in mammalian skeletal muscles^57^.

Ion	Concentration [mM]		Nernst potential [mV]
	Inside	Outside	
Na^+^	12	145.0	+67
K^+^	155	4.0	−98
Ca^2+^	10^−4^	1.5	+129
Cl^−^	4	120.0	−90

1 M = mol L^−1^.
